# A High-Order EMSIW MIMO Antenna for Space-Constrained 5G Smartphone

**DOI:** 10.3390/s21248350

**Published:** 2021-12-14

**Authors:** Sayyed A. Ali, Mohd Wajid, Mohammed Usman, Muhammad S. Alam

**Affiliations:** 1 Department of Electronics Engineering, Zakir Husain College of Engineering and Technology, Aligarh Muslim University, Aligarh 202002, India; sayyedarifali@zhcet.ac.in; 2 Department of Electrical Engineering, King Khalid University, Abha 61411, Saudi Arabia; omfarooq@kku.edu.sa; 3 Department of Electrical Engineering, College of Engineering, Imam Muhammad Ibn Saud Islamic University, Riyadh 11432, Saudi Arabia; malam@imamu.edu.sa

**Keywords:** 5G smartphone, eighth-mode substrate integrated waveguide (EMSIW), multiple-input multiple-output (MIMO) antenna, sub-6 GHz band, specific absorption rate (SAR)

## Abstract

This paper proposes a high-order MIMO antenna operating at 3.5 GHz for a 5G new radio. Using an eighth-mode substrate integrated waveguide (EMSIW) cavity and considering a typical smartphone scenario, a two-element MIMO antenna is developed and extended to a twelve-element MIMO. These MIMO elements are closely spaced, and by employing multiple diversity techniques, high isolation is achieved without using a decoupling network. The asymmetric EMSIW structures resulted in radiation pattern diversity, and their orthogonal placement provides polarization diversity. The radiation characteristics and diversity performance are parametrically optimized for a two-element MIMO antenna. The experimental results exhibited 6.0 dB and 10.0 dB bandwidths of 250 and 100 MHz, respectively. The measured and simulated radiation patterns are closely matched with a peak gain of 3.4 dBi and isolation ≥36 dB. Encouraged with these results, higher-order MIMO, namely, four- and twelve-element MIMO are investigated, and isolation ≥35 and ≥22 dB are achieved, respectively. The channel capacity is found equal to 56.37 bps/Hz for twelve-element MIMO, which is nearly 6.25 times higher than the two-element counterpart. The hand and head proximity analysis reveal that the proposed antenna performances are within the acceptable limit. A detailed comparison with the previous works demonstrates that the proposed antenna offers a simple, low-cost, and compact MIMO antenna design solution with a high diversity performance.

## 1. Introduction

The 5G new radio (NR) technology has paved the way for enhanced mobile broadband (eMBB) services, which allow the exchange of high data rates [1], and provides an opportunity for developing antennas for such applications, operating in the sub-6 GHz band (i.e., FR1) and millimeter-wave (mmWave) band (i.e., FR2). The FR1 band provides broader coverage and lower data rate, whereas the FR2 offers higher data rates but a limited coverage area. A detailed literature review [1,2] suggests that the most widely used bands for 5G NR are 3.5 GHz for the FR1 and 28 GHz for FR2. Hand-held devices, such as mobile phones and tablets, generally utilize eMBB services, and therefore, the antennas for these services need to fulfill various requirements [2]. The critical requirement, among others, is high data rates; therefore, a high order MIMO is indispensable in these devices operating in FR1/FR2 bands to achieve data rates up to 20 Gbps [2]. Furthermore, due to the limited availability of space, compact size antennas and their placement on a space-constrained smartphone scenario are required to ensure a low-cost solution [3].

In recent years, substrate integrated waveguide (SIW) technology [4] emerged as a new planar technology with its cost comparable to microstrip technology, at the same time offering high power handling capability [3]. In the SIW, via walls provide high isolation with other radio circuit components [3]; however, designing a compact MIMO antenna in SIW technology is challenging due to its geometrical constraints [5]. Therefore, most of the MIMO antennas are developed using microstrip technology, and very few works are reported in the literature using SIW technology [6]. Moreover, with the advent of hybrid boundary techniques [7,8], miniaturized SIW cavities have been developed [9,10,11,12,13,14,15,16,17,18] and used for compact SIW antennas. They include half-mode SIW (HMSIW), quarter-mode SIW (QMSIW), and eighth-mode SIW (EMSIW) [12,13], which are used to develop compact MIMO antennas. In [9,10,11,12,13], the MIMO antenna utilized HMSIW, where, except [11], all others used rectangular cavity, whereas [11] employed a circular cavity. Furthermore, these MIMO antennas used slots in the cavity except [13], where a rectangular patch antenna was parasitically excited using HMSIW.

The QMSIW was used to develop MIMO antennas [14,15,16], where the bandwidth enhancement [14,16] and multiband [15] operations were adopted in the designs but at the expense of reduced isolation as compared to the FMSIW [6] or HMSIW [9,10,11,12,13]. However, the sizes of the FMSIW, HMSIW, and QMSIW designs are large compared to the microstrip antennas. To the best of the authors’ knowledge, limited studies [5,17,18,19,20] are available for antenna design using EMSIW, where other reported works are MIMO designs except [19]. Four closely spaced EMSIW elements were used [17], but the gain achieved was very low (−3 dBi). Similarly, the four-element EMSIW MIMO antenna [20] reported a gain of 3.1 dBi with good isolation. At the same time, in [5] and [18], two-element EMSIW antennas were reported, operating in the sub-6 GHz band. In earlier work [5], a rectangular slot was used in the ground plane for decoupling purposes to achieve high isolation of 30 dB, and in the latter case [18], no extra decoupling network was used, and a minimum isolation of 18.5 dB was achieved. The SIW MIMO antennas [9,10,11,12,13,14,15,16,17,18,19,20] were developed without considering the space-constrained smartphone scenario, where edges and corners are the only space available for antenna placement [21,22,23,24,25,26,27,28,29]. Therefore, it requires a careful antenna design and placement strategy to fulfill space-related constraints in such a scenario. As per the authors’ knowledge, the proposed work is the first SIW MIMO antenna, considering a realistic space-constrained scenario in smartphones.

Furthermore, keeping mutual coupling and envelope correlation coefficient (ECC) between antennas below threshold levels [30] is more challenging, especially in a multi-antenna environment. The MIMO antennas utilized the SIW via walls [6,11,12], pattern diversity [9], and polarization diversity [13] to achieve isolation; however, in [5], neither via wall nor pattern or polarization diversity was used. Therefore, an extra decoupling network (DN) was employed at the cost of introducing additional design complexity. In order to avoid DN and to achieve high isolation, there is a need to utilize multiple diversity techniques in closely spaced MIMO antennas. These aspects are incorporated in the proposed work.

An EMSIW element operating at 3.5 GHz and its two-element MIMO antenna is first studied and achieved most of the critical requirements of 5G NR, such as the gain, bandwidth, and diversity. The simulation results achieved are corroborated by measuring the proposed two-element MIMO antenna by developing its prototype and found to satisfy the radiation and bandwidth requirements of the 5G NR. Furthermore, multipath fading is investigated using mean effective gain (MEG), and results were found within limits [30]. By incorporating multiple diversity techniques, supported by detailed parametric analysis, isolation is optimized without using any complex decoupling network (DN) in the proposed design. Encouraged by the two-element MIMO antenna results, the concept is extended to detailed simulation study of high-order MIMO antenna, namely, four and twelve-element MIMO antenna for smartphones. In this highly space-constrained scenario, closely spaced antennas are strategically placed, and high isolation performance is realized. Furthermore, the channel capacity simulation study [21] demonstrated the proposed antenna multiplexing capability using the standard channel models. The user’s hand analysis and the SAR study (by considering head phantom) are carried out to validate that the proposed design of the antenna is for smartphones.

The remainder of the paper is organized as follows. Section 2 discusses the methodology involved for twelve-element EMSIW MIMO antenna design. Further, this section covers the parametric analysis to enhance the MIMO diversity performance and systematic development approach of the proposed antenna from a low-order to a high order. Section 3 elaborates the proposed two-element MIMO antenna prototype and compares the simulated and measured radiation and diversity results. Furthermore, advancing the design concept, a diversity investigation of four-element and twelve-element MIMO antennas is carried out, including evaluating channel capacity considering the CBC and Winner II channel models, and a hand mode and SAR study is performed. Finally, the significance of the proposed antenna is established through a comprehensive performance comparison, and finally, conclusions are drawn in Section 4.

## 2. Proposed MIMO Antenna

### 2.1. Antenna Geometry

**Figure 1:**
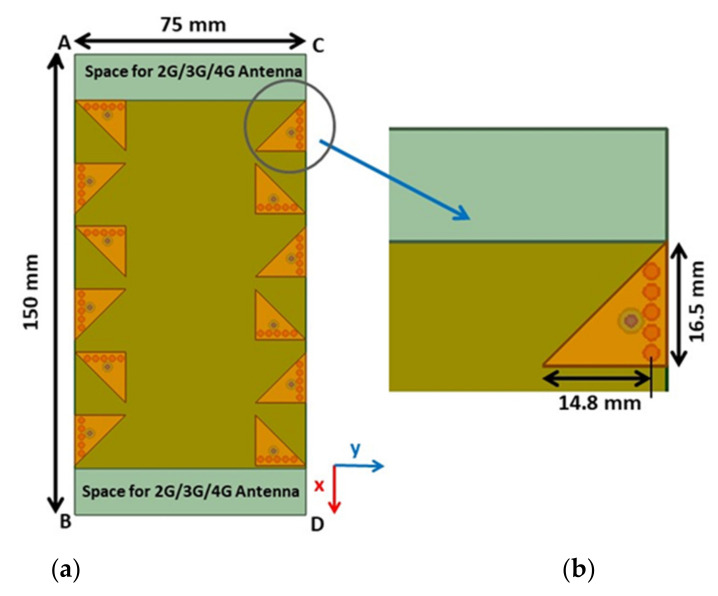
(**a**) The perspective view of the proposed twelve-element MIMO antenna (**b**) Structural details of the EMSIW unit antenna element, i.e., Ant 3 with L_r_ = 14.8 mm, W_c_ = 16.5 mm, d = 2 mm, s = 2.7 mm, P_1_ = 5.95 mm, and P_2_ = 4.8 mm.

The proposed MIMO antenna geometry is shown in Figure 1a. In this figure, twelve antenna elements are placed along the two long edges AB and CD of the 1.6 mm thick double-sided FR4 substrate PCB with a relative permittivity of 4.4 and a loss tangent of 0.02. The typical size of the substrate PCB is 150 × 75 × 1.6 mm [23,24], where two rectangular regions of 15 × 75 mm on the short edges AC and BD of the PCB are left for accommodating 2G/3G/4G antennas. Figure 1b depicts the geometry details of the unit EMSIW antenna element, which is used to build the proposed twelve-element MIMO antenna.

**Figure 2:**
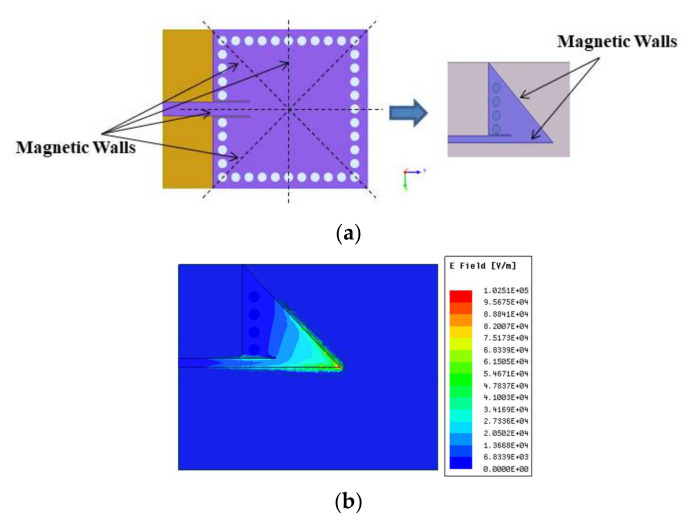
(**a**) Symmetrical cutting of FMSIW along the quasi-magnetic walls (dotted lines), and subsequently obtaining the eighth-mode SIW (EMSIW) antenna element. (**b**) Electric field distribution in the EMSIW antenna for the dominant mode.

Inspired by the work [31], EMSIW was evolved using the FMSIW cavity, as shown in Figure 2a. Figure 2b shows the dominant mode electric field distribution in the EMSIW antenna. All simulation results in the proposed work were carried out using Ansys HFSS [32].

### 2.2. Single EMSIW Antenna

**Figure 3:**
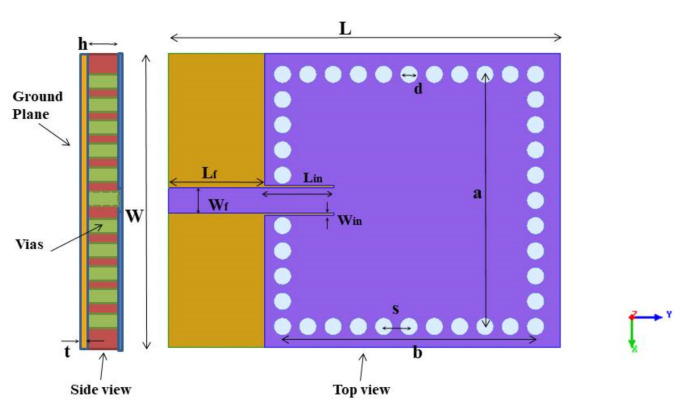
Structure of the FMSIW cavity with the side and top views.

In the antenna design process, a square-shaped full-mode SIW (FMSIW) cavity operating at 3.5 GHz was designed and simulated on FR4 substrate, as shown in Figure 3. The resonance frequency for different cavity modes *f_mn_*_0_ is expressed as [7]

(1) $$f_{mn0}=\frac{c}{2\pi \sqrt{\mu \varepsilon_{r}}}\sqrt{{\left(\frac{m\pi }{L_{eff}}\right)}^{2}+{\left(\frac{n\pi }{W_{eff}}\right)}^{2}}$$

(2) $$L_{eff}=a-1.08\frac{d^{2}}{s}+0.1\frac{d^{2}}{a}$$

(3) $$W_{eff}=b-1.08\frac{d^{2}}{s}+0.1\frac{d^{2}}{b}$$

where *a* and *b* are the length and width of the SIW cavity (*a = b* in the proposed study); *d* and *s* are the via diameter and the pitch of the vias, and *ε_r_* is the relative permittivity of the substrate. The SIW cavity behaves identically to a conventional metallic cavity when *d/s* ≥ 0.5 and *d/λ_o_* ≤ 0.1 [7]. Furthermore, conditions such as *d* < *s* < 2 *d* must be satisfied to avoid the bandgap effect [7], which arises due to periodic structures, i.e., vias of SIW. Then, the various dimensions of the square cavity were determined using Equations (1)–(3) and further optimized in Ansys HFSS [32], and their optimized values are summarized in Table 1. Since there is a large SIW width ratio to height, only TE_mn0_ modes with *p* = 0 exist in the SIW resonator [7].

**Figure 4:**
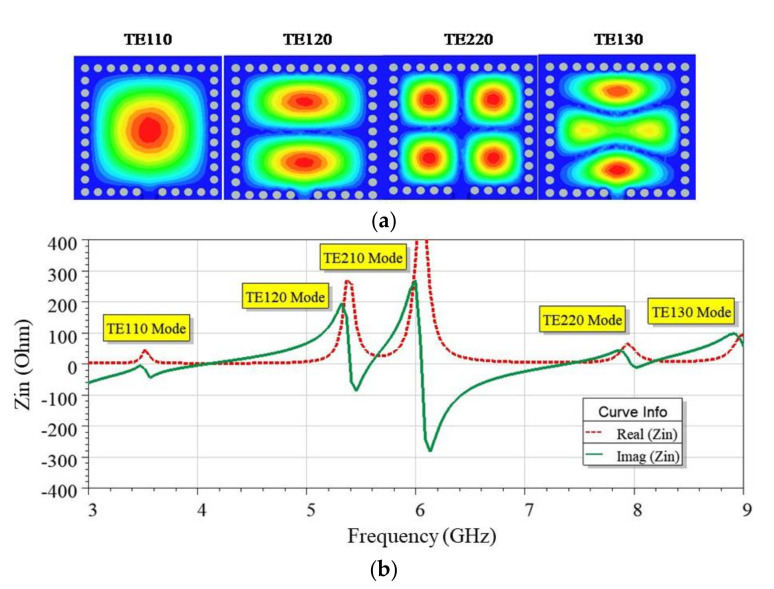
Plot for different modes existing in the FMSIW cavity, (**a**) field distribution and (**b**) real and imaginary parts of input impedance Z_in_.

The Eigenmode analysis using HFSS revealed the different modes existing in the FMSIW cavity. The electric field distribution in dominant mode TE_110_ at 3.56 GHz and higher-order modes, i.e., TE_120_, TE_220_, and TE_130_, are plotted in Figure 4a.

Subsequently, input impedance (Z_in_) variation in various modes in the cavity is shown in Figure 4b, when a 50 Ω microstrip line feeds the cavity. In the modes TE_110_, TE_220_, and TE_130_, the FMSIW cavity showed good matching characteristics, i.e., re(Z_in_) ≅ 50.0 Ω, and im(Z_in_) ≅ 0.0. However, in this work, an antenna was developed operating in dominant mode. As shown in Figure 2a, symmetrical field longitudinal planes exist in the FMSIW in the dominant mode, which were used to construct the EMSIW cavity. These longitudinal planes are quasi-magnetic walls where the electric field is the maximum and the magnetic field is zero [31]. If the cut was made along these lines, the field patterns in the dominant mode were not perturbed (see Figure 2b). The resulting cavity so developed was called EMSIW cavity, as the two open edges, behaving as a radiating slot. The resonating frequency in the dominant mode (*fc*_110_) for EMSIW was given as [31]:

(4) $$f_{C_{110}}=\frac{\sqrt{2}c}{2a\sqrt{\varepsilon_{r}}}$$

**Figure 5:**
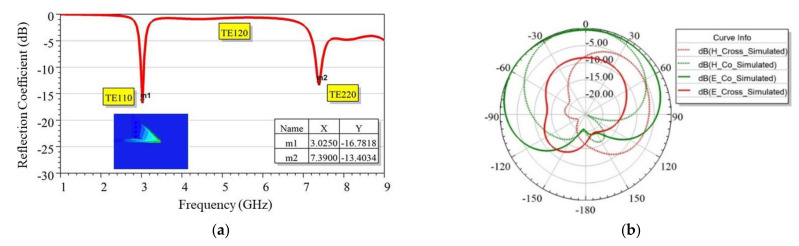
Simulated performances of a single EMSIW antenna. (**a**) Reflection coefficient (**b**) Co-pole and cross-pole radiation pattern plots in E-plane (xz, ϕ = 0°) and H-plane (yz, ϕ = 90°) at 3.025 GHz.

**Figure 6:**
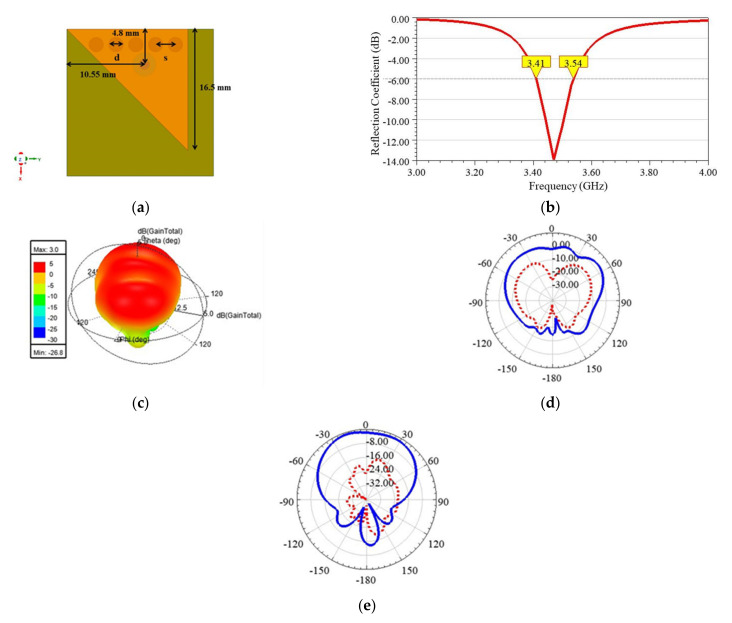
Results of EMSIW antenna excited by coaxial probe feed. (**a**) Geometry, (**b**) reflection coefficient, (**c**) 3D polar plot and co-pole (blue) and cross-pole (red) radiation pattern plots in (**d**) E-plane (xz, ϕ = 20°) and (**e**) H-plane (yz, ϕ = 110°) at 3.44 GHz.

The *fc*_110_ was observed at 3.025 GHz when a single EMSIW element was used, as shown in Figure 5a. A shift in operating frequency from 3.56 GHz in FMSIW to 3.025 GHz in the EMSIW was primarily due to the fringing effect around two quasi-magnetic walls, which further helped to miniaturize the size of the antenna. Both co- and cross-pole normalized radiation patterns for a single EMSIW element were plotted in E and H planes, as shown in Figure 5b. The EMSIW antenna was observed to give a broadside radiation pattern; however, its main lobe direction was tilted by around θ = −20° [31]. The pattern suggests that the antenna was linearly polarized. The simulated gain at 3.025 GHz was found equal to 2.88 dBi, whereas the impedance bandwidth taken at −6 dB and −10 dB of reflection coefficient (S11) was found equal to 140 MHz and 80 MHz, respectively. In the smartphone scenario, −6 dB bandwidth is an acceptable criterion [21]. Therefore, it is concluded that the performances of the EMSIW antenna are good enough for use in 5G NR, which requires a minimum BW of 100 MHz [2]. These results were generated using microstrip feed; however, to save the precious circuit footprint on PCB, the microstrip feed was replaced by coaxial feed (see Figure 6a) to excite EMSIW antenna in the subsequent discussion. The resonating frequency of the EMSIW antenna was set at 3.44 GHz. It was observed from Figure 6b that −6 dB bandwidth is 130 MHz, whereas Figure 6c reveals that the E-plane and H-plane exist at ϕ = 20° and ϕ = 110°, respectively. This shifting of the plane is due to the asymmetric structure of the EMSIW antenna. Accordingly, E-plane and H-plane radiation patterns are plotted in Figure 6d,e, respectively. In the E-plane, the cross-pole component is less than −10 dB, and in the H-plane, it is less than −14 dB. Slightly increased cross-pole levels can be attributed to the use of coaxial feed [5]. The EMSIW antenna with coaxial feed is now used as a basic building block to develop higher-order MIMO designs in the following sections.

### 2.3. Parametric Study of Two-Element MIMO Antenna

In order to develop a high order twelve-element MIMO antenna array using EMSIW, a parametric study was performed on a two-element MIMO antenna to optimize the diversity performance of the closely spaced multiple antennas. Before placing two elements closely, first, polarization and radiation characteristics of a single EMSIW element were investigated because the knowledge acquired would help to achieve high isolation and diversity performances when more than two elements are used for developing a higher-order MIMO antenna. The proposed antenna design uses multiple diversity techniques, viz., polarization, pattern, and spatial, to achieve high isolation without using a complex DN [33,34].

As shown in Figure 5b, the EMSIW main lobe of radiation was tilted due to the asymmetric structure of the EMSIW antenna. Therefore, it helps achieve pattern diversity [35], which is not possible to get from symmetrical SIW structures. The two features of tilted radiation and linear polarization are utilized in the developed MIMO antenna to achieve pattern and polarization diversity.

**Figure 7:**
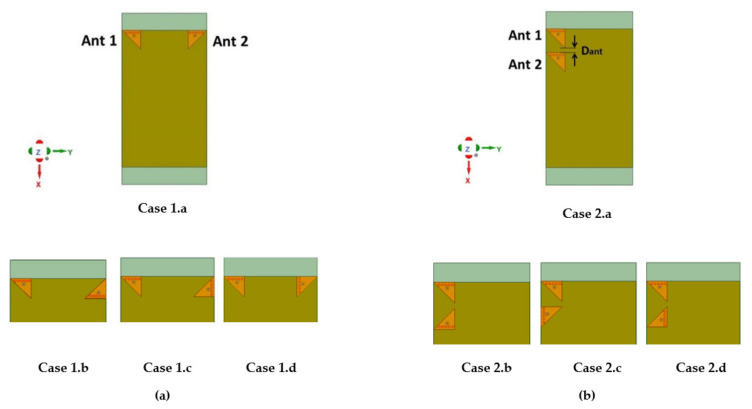
Different antenna arrangement for two-element MIMO antenna configuration in (**a**) edge scenario and (**b**) adjacent scenario.

In the parametric study, two scenarios are considered. Figure 7a shows the first edge scenario and Figure 7b shows the second, the adjacent scenario. In the edge scenario, the antennas are placed on opposite edges, whereas in the adjacent scenario, the two antennas are placed on the same edge close to each other. In each scenario, four cases exist for four different antenna orientations. All these cases, namely, Case 1.a, Case1.b, Case1.c, and Case 1.d and Case 2.a, Case 2.b, Case 2.c, and Case 2.d were included in the parametric study to optimize their performance and subsequently use these results for optimized performance for the four-element MIMO antenna design (see Section 2.4).

#### 2.3.1. Case 1—When Antenna Elements Are Placed at the Edges

In the first part of the study, an edge scenario with four different cases was investigated, as shown in Figure 7a. In this scenario, two EMSIWs were placed on the two opposite long edges of the PCB with four different mutual orientations as designated by Case 1.a, Case 1.b, Case 1.c, and Case 1.d.

**Figure 8:**
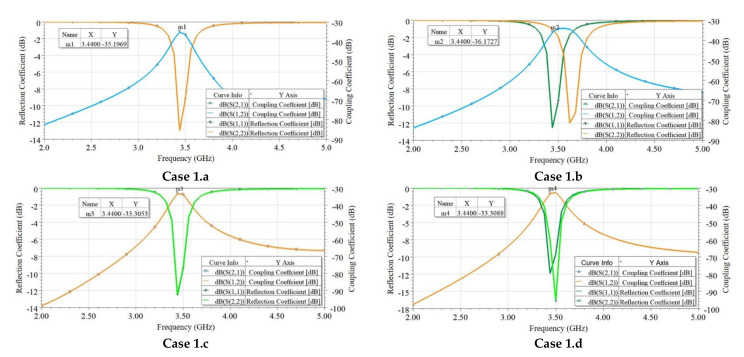
Simulated reflection coefficient (*S_ii_*) and coupling coefficient (*S_ij_*) plots for four cases in edge scenario.

**Figure 9:**
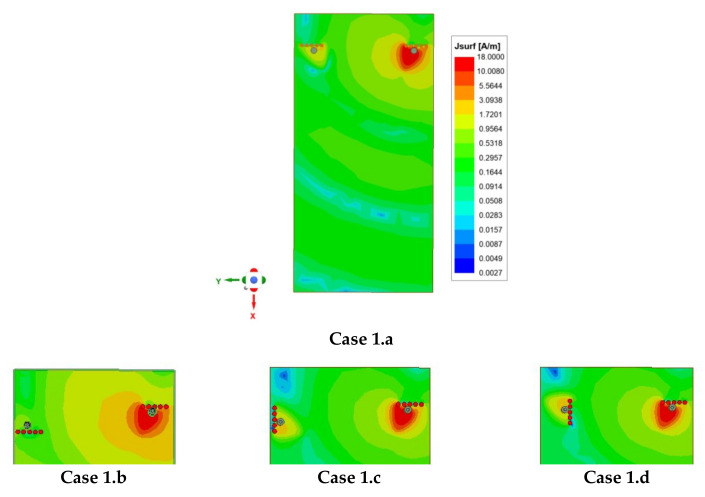
Surface current density distribution on the ground plane (reverse side of PCB) for two-element MIMO antenna designs **Case 1.a** to **Case 1.d** at 3.43 GHz, when Ant 1 is excited and Ant 2 is terminated with 50 Ω.

The reflection (S_ii_) and coupling (S_ij_) coefficients were plotted in all four cases, as shown in Figure 8. Similarly, surface currents on the ground plane were plotted in each case, as shown in Figure 9. As observed from Figure 8, coupling in Case1.b is minimum (−36 dB), which corresponds to the lower value of coupling surface current flowing from Ant 1 towards Ant 2, as shown in Figure 9 (Case 1.b). However, the resonating frequency of Ant 2 in Case 1.b is slightly shifted from the desired frequency, which is due to the reduced fringing fields as the resonating side of Ant 2 is on the extreme edge of the PCB. Therefore, the better choice is Case 1.a because it offers comparable isolation (35 dB) with Case 1.b and more stability in operating frequencies for both antennas.

**Figure 10:**
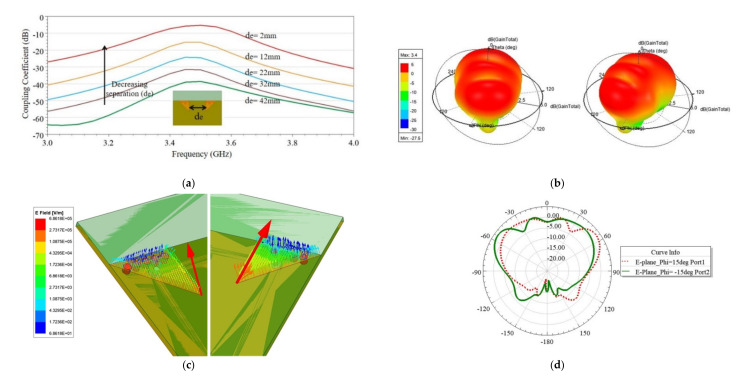
Various investigations carried out for two-element MIMO antenna in Case 1.a: (**a**) Parametric variation of mutual coupling when the antenna separation (de) is varied; (**b**) pattern diversity between the two EMSIW antenna elements is visible in polar plots; (**c**) E-field vectors orientation, and (**d**) E-plane cuts for the two EMSIW antennas at 3.43 GHz.

In all the four cases, namely, Case 1.a–1.d, it is observed that isolation was >30 dB. Hence, relative rotation/flipping of Ant 2 with respect to Ant 1 did not much affect the isolation. Therefore, it is concluded that there is a minimal role of pattern or polarization diversity; only spatial diversity is responsible for achieving high isolation. This is further confirmed from the investigation performed for spatial antenna separation (de). Figure 10a shows the variation of antenna coupling with the separation (de). As observed from Figure 10a, by decreasing the separation, coupling increased. More specifically, when the antennas were placed at the PCB edges, at a distance of de = 42 mm, approximately equal to half of the operating wavelength (42.55 mm), it resulted in maximum isolation. Thus, spatial diversity was mainly responsible for high isolation. Additionally, pattern diversity exists between the antennas. The 3D polar radiation and E-plane plots are shown in Figure 10b,d, respectively, and E-field vector variations are plotted in Figure 10c. From these plots, it is obvious that both the antennas radiated in different directions; the reason is the asymmetric structure of the EMSIW antenna, which is inherently responsible for diversified beams. No additional mechanism is required in the proposed design to tilt the beams. To demonstrate the proposed design concept in this work, the antenna discussed in Case 1.a was validated by developing its prototype, and measured results are discussed in Section 3.

#### 2.3.2. Case 2—When Antenna Elements Are Placed Adjacently

**Figure 11:**
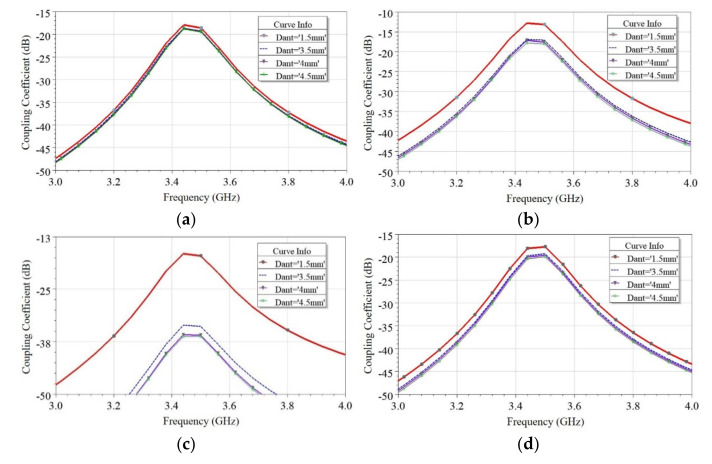
Effect of antenna separation (*D_ant_*) on coupling (S_ij_) (**a**) Case 2.a, (**b**) Case 2.b, (**c**) Case 2.c, and (**d**) Case 2.d.

**Figure 12:**
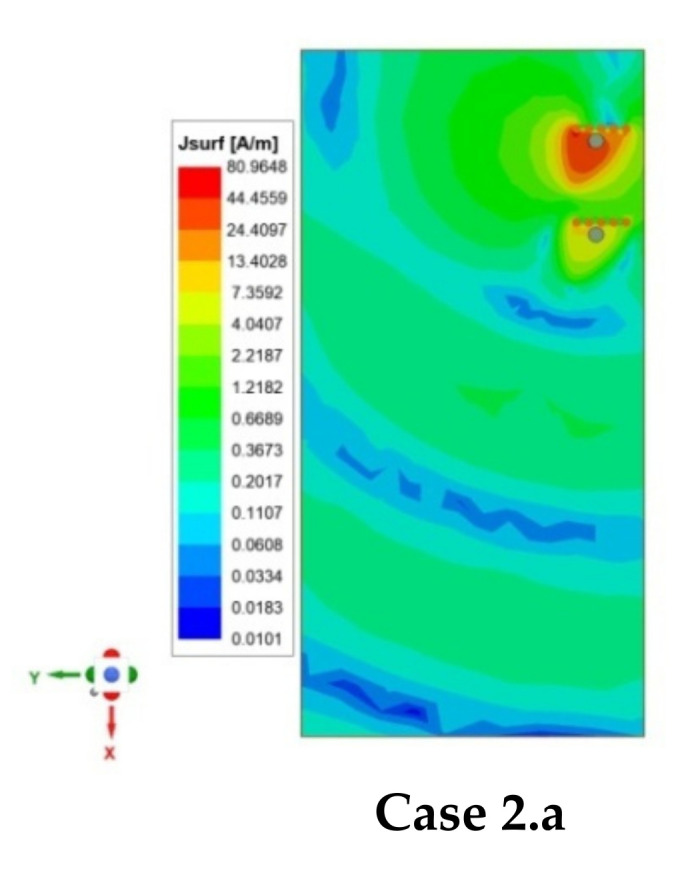

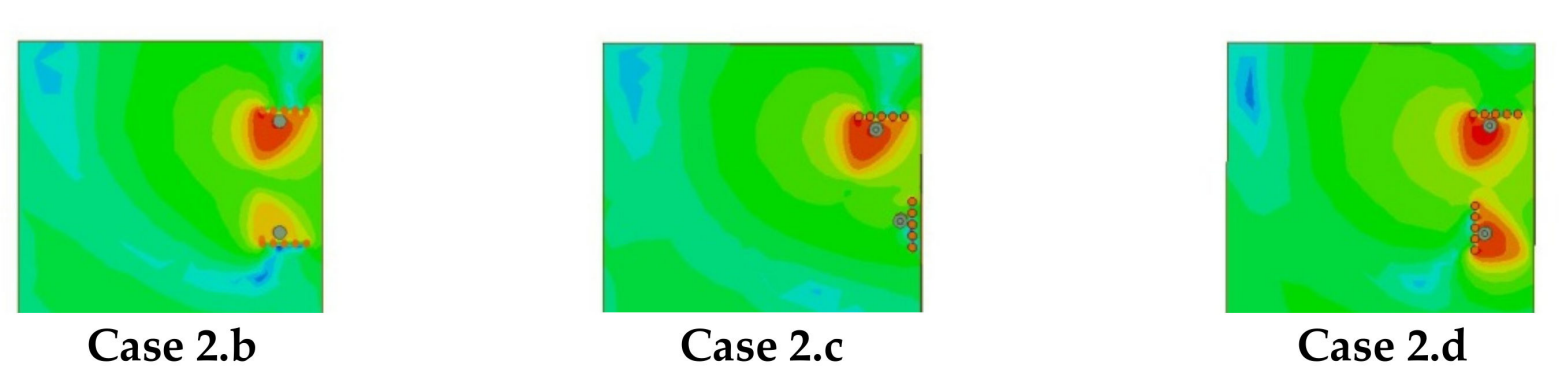
Surface current density distribution on the ground plane for two-element MIMO antenna designs in different cases, namely, **Case 2.a** to **Case 2.d** at 3.43 GHz for *D_ant_* = 4mm, when Ant 1 is excited and Ant 2 is terminated with 50 Ω.

Similarly, four cases, namely, Case 2.a to Case 2.d for the adjacent scenario, is discussed, as shown in Figure 7b. In this scenario, two antenna elements are closely placed on the same edge of the PCB with different mutual orientations, and the separation, *D_ant_*, between the antennas is optimized to maximize the isolation for each case. Figure 11 shows the effect of *D_ant_* on coupling (S_ij_), and surface current density distribution is plotted in Figure 12 for all four cases. It is a known fact that if antennas are closely placed along the same edge, there is the possibility of high coupling. Many researchers in the past developed such closely spaced MIMO antenna, and the coupling issue was reduced by using a decoupling network (DN) [5,23,30,33,34]. However, the DN complicates the antenna design and adds complexity. Multiple diversity techniques are utilized in the proposed design to achieve significantly high isolation to avoid such decoupling complex structures. Various diversity techniques, e.g., pattern and orthogonal polarization diversity, are used to achieve high isolation in the proposed design. Table 2 summarizes the mutual diversities existing between the two antennas in all four cases. In Case 2.a and Case 2.b, neither pattern nor orthogonal polarization diversity exists due to similar orientation of resonating edge of the two antennas, although in Case 2.a slightly better isolation is obtained due to isolation created by via wall. Furthermore, in Case 2.c and Case 2.d, both pattern and orthogonal polarization diversity exist but Case 2.c only provides isolation (S_21_) >35 dB at an optimized distance *D_ant_* = 4 mm (0.045 *λ*) (see Figure 11). This is further confirmed from the surface current plot on the ground plane shown in Figure 12, which shows the minimum surface current flowing from Ant 1 towards Ant 2. Hence, the minimum electromagnetic coupling is observed in Case 2.c. Suppose Case 2.c and 2.d are compared. In that case, the main reason behind poor isolation in Case 2.d is due to the closeness in two ground current regions (see Figure 12). In contrast, in Case 2.c, the two grounds are separated by the radiating slot of Ant 2; thus, isolation is developed between the feeding ports. Therefore, this guideline will help in arranging a higher number of antennas closely. Hence, without using any DN, high isolation (>35 dB) is achieved in closely spaced (0.045 *λ*) antennas by utilizing multiple diversity techniques.

### 2.4. Four-Element MIMO Antenna

**Figure 13:**
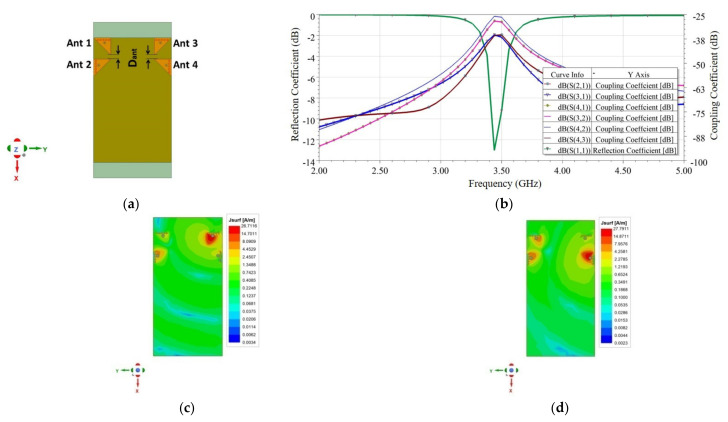
Various investigations carried out for four-element MIMO antenna. (**a**) Geometrical details, (**b**) simulated S-parameters of optimized four-element EMSIW MIMO antenna and its surface current distribution at 3.43 GHz, (**c**) Ant 1 excited, and (**d**) Ant 2 excited when all other antennas are matched.

In Section 2.3, two optimized edge (Case 1.a) and adjacent (Case 2.c) scenario designs were obtained; subsequently, a four-element EMSIW MIMO antenna was developed by combining both the cases, as shown in Figure 13a. Its simulated S-parameter details are plotted in Figure 13b. Closely spaced antennas Ant 1 and Ant 2 and Ant 3 and Ant 4 are highly isolated with more than 35 dB without the use of any decoupling network (DN). Surface current density distribution further confirms the high isolation achieved. Simulated results demonstrated that the proposed two-element MIMO antenna provides firm ground to develop a high-order MIMO, including its diversity performance, as discussed in Section 3.

### 2.5. Twelve-Element MIMO Antenna

**Figure 14:**
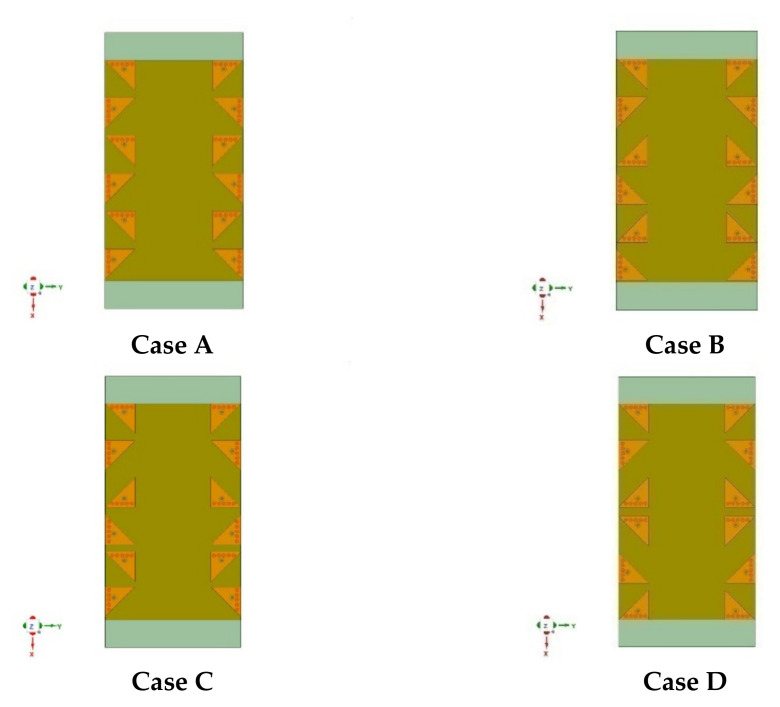
Four different cases, i.e., **Case A**, **Case B**, **Case C**, and **Case D**, of antenna arrangement for twelve-element EMSIW MIMO antenna design.

The design concept discussed above for the four-element MIMO design was further extended to develop a high-order twelve-element EMSIW MIMO antenna, considering a smartphone scenario. The four-element MIMO design was replicated three times on the PCB to develop a twelve-element MIMO antenna (Case A), as shown in Figure 14. However, three more cases were parametrically studied, e.g., Case B, Case C, and Case D, as shown in Figure 14. Different antenna orientations were used in these designs to obtain an optimized MIMO antenna structure. The antenna numbering convention is the same as followed in Figure 1.

**Figure 15:**
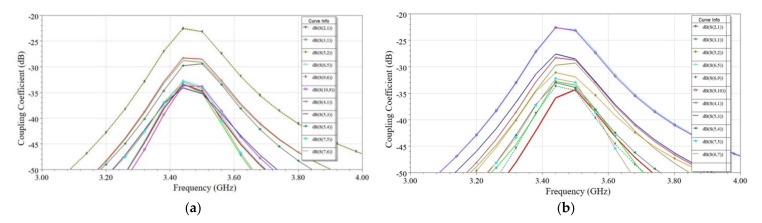

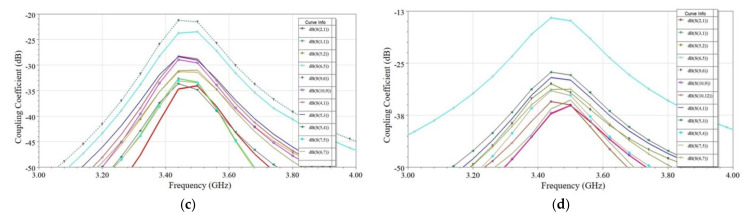
(**a**,**b**,**c**,**d**) Simulated coupling coefficients for various cases, i.e., Case A, Case B, Case C, and Case D, of twelve-element MIMO antenna designs.

Figure 15 shows the variation of coupling coefficients in all four cases. Coupling for antenna pairs on the same edge, namely, Ant 1 and Ant 2, Ant 2 and Ant 5, Ant 5 and Ant 6, Ant 6 and Ant 9, and Ant 9 and Ant 10 are plotted in Figure 15 along with adjacent antenna pairs, namely, Ant 1 and Ant 4, Ant 3 and Ant 5, Ant 4 and Ant 5, Ant 5 and Ant 7, and Ant 6 and Ant 7. Due to symmetry, the remaining antenna pairs were skipped for brevity. It is observed from Figure 15 that in Case A, except for antenna pairs Ant 2 and Ant 5 and Ant 9 and Ant 10, all other antenna pairs’ isolation was above 28 dB. For these two pairs of antennas, the coupling was found around −22 dB.

Similarly, in Case B, the antenna pairs Ant 5 and Ant 6 and Ant 9 and Ant 10 showed the coupling at the same −22 dB level. However, out of these two cases, Case A was the better one for realization because antenna pair Ant 9 and Ant 10 was near the corner side of PCB, hence, more prone to further distortion due to coupling incurred by the hands of smartphone users [21,22]. In Case C, antenna pairs Ant 5 and Ant 6 and Ant 6 and Ant 9 were coupled with −21 dB and −24 dB levels, whereas in Case D, antenna pair Ant 5 and Ant 6 coupled with a level of −14 dB, making it unsuitable for practical application. Hence, the coupling analysis revealed that Case A is the most practical arrangement for a twelve-element MIMO antenna design. Furthermore, its diversity analysis is discussed in Section 3.

## 3. Results, Discussions, and Performance Comparisons

### 3.1. Two-Element MIMO Antenna

**Figure 16:**
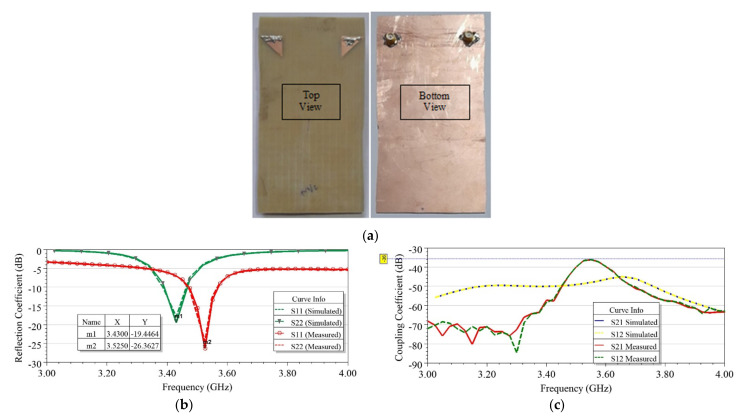
(**a**) Fabricated prototype of two-element MIMO antenna in Case 1.a, using the EMSIW antenna element (top and bottom view); (**b**) reflection coefficient (S_ii_), and (**c**) coupling coefficient (S_ij_).

To validate the simulation results, the two-element EMSIW MIMO antenna discussed in Case 1.a was fabricated and the results measured. The fabricated prototype is shown in Figure 16a. Both EMSIW antenna elements were excited using the coaxial feed for testing purposes. However, suitable transitions could be used to integrate the antenna with the radio circuit for practical implementations in the smartphone scenario, as discussed in [36]. Copper rivets of 2 mm diameter were inserted in the via holes of the EMSIW and then soldered to cover the via to develop the MIMO antenna. The antenna S-parameters were measured using Agilent’s PNA-L N5234A network analyzer, and radiation patterns were recorded in an anechoic chamber manufactured by Rhode & Schwarz using HF907 double-ridged waveguide horn antenna. The various performances for the proposed MIMO antenna, such as reflection coefficient (S_11_), gain, radiation patterns in E and H planes, and diversity performances, including isolation, ECC, and mean effective gain (MEG), were measured and compared with their simulated value.

**Figure 17:**
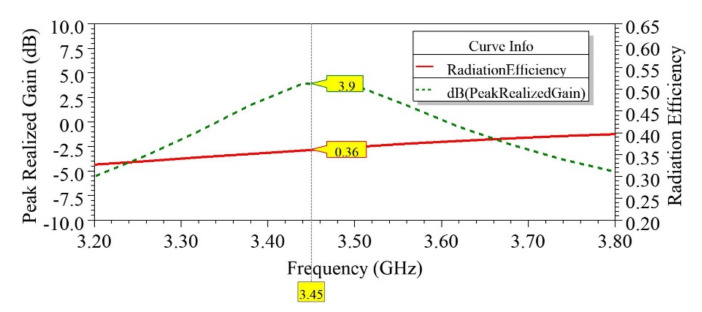
Simulated peak realized gain and radiation efficiency of the two-element MIMO antenna.

**Figure 18:**
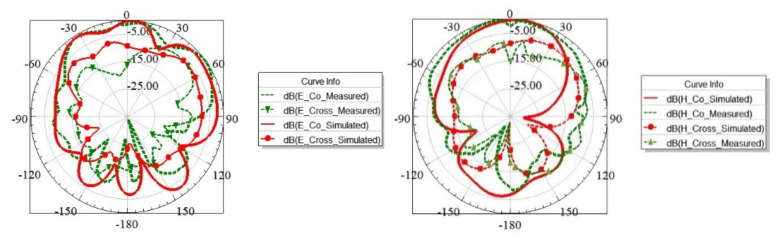
Simulated and measured co and cross-pole normalized radiation patterns at 3.525 GHz in free space in E-plane (xz, ϕ = 0°) and H-plane (yz, ϕ = 90°) of Ant 1 when Ant 2 is matched to 50 Ω.

As observed from the results shown in Figure 16b, the MIMO antenna’s measured impedance bandwidth was found equal to 250 MHz at −6dB@S_11_ and 100 MHz at −10dB@ S_11_, when the MIMO antenna was resonating at 3525 GHz. Due to fabrication errors, a slight shift in resonance frequency of 0.095 GHz was observed from the simulated result. High isolation (S_12_/S_21_) of >36 dB between the antenna elements was confirmed by the measured results, as shown in Figure 16c. The simulated and measured peak realized gains of the proposed antenna were 3.9 dBi @3.45 GHz and 3.4 dBi @3.525 GHz, respectively. A minimum simulated gain of 3 dBi over the BW of interest and radiation efficiency (η) of nearly 36% was realized, as shown in Figure 17. Efficiency could be further enhanced by using low-loss dielectric substrates [8]. The simulated and measured radiation patterns inside the anechoic chamber of the fabricated prototype antenna were recorded and normalized, and subsequently, the results were plotted. Figure 18 shows the broadside radiation pattern of the first EMSIW antenna, marked as Ant 1, when the second antenna, Ant 2, was matched to 50 Ω. The co-polar and cross-polar components of the E and H fields are displayed in Figure 18, suggesting that the main lobe is tilted by approximately θ = −30° from the broadside directions in the E-plane with a cross-polar level of 10 dB below the co-polar component.

**Figure 19:**
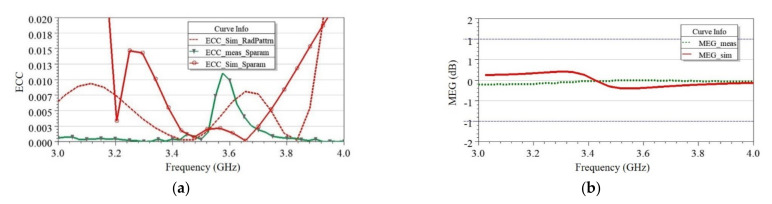
Simulated and measured (**a**) ECC determined using radiation pattern and S-parameters and (**b**) mean effective gain (MEG) evaluated within the limit of ±1 dB for the proposed two-element MIMO antenna.

Furthermore, the MIMO diversity performance of the developed antenna was evaluated using the following equations [30]:

(5) $$ECC=\frac{{\left|\iint_{4\pi }^{}\left[\vec{\Psi_{1}}\left(\theta ,\varphi \right)*\vec{\Psi_{2}}\left(\theta ,\varphi \right)\right]d\Omega \right|}^{2}}{\iint_{4\pi }^{}{\left|\vec{\Psi_{1}}\left(\theta ,\varphi \right)\right|}^{2}d\Omega \iint_{4\pi }^{}{\left|\vec{\Psi_{2}}\left(\theta ,\varphi \right)\right|}^{2}d\Omega }$$

(6) $$ECC=\frac{{\left|S_{ii}^{*}S_{ij}+S_{ji}^{*}S_{jj}\right|}^{2}}{\left(1-{\left|S_{ii}\right|}^{2}-{\left|S_{ji}\right|}^{2}\right)\left(1-{\left|S_{jj}\right|}^{2}-{\left|S_{ij}\right|}^{2}\right)}$$

where $\vec{\Psi_{i}}\left(\theta ,\varphi \right)$ is the three-dimensional radiation pattern of the antenna when the *i*th port is excited and Ω is the solid angle. The asterisk is the Hermitian product operator. Since the two antennas were spatially and pattern diverse, high isolation was achieved, as discussed in the previous section. Moreover, the proposed MIMO antenna reached significantly low ECC < 0.013 over the frequency band of interest, as shown in Figure 19a, which included the simulated and measured ECC (S-parameters) and simulated ECC (radiation pattern). All values of ECCs were below 0.013 at resonating frequency 3.525 GHz. The proposed antenna in this work did not utilize any extra decoupling networks (DN) to achieve low mutual coupling and ECC, thereby reducing the design complexity. The high isolation achieved met the minimum required isolation of value 15 dB for 5G new radio [30].

In the wireless fading channel environment, mean effective gain (MEG) provides the amount of power received with reference to an isotropic antenna. The expression for MEG reported in [35] was used, and it was found within the ±1 dB range, as shown in Figure 19b. Hence, obtained ECC and MEG values indicate that the proposed MIMO antenna is capable of robust performances under the fading channel environment [35].

### 3.2. Four-Element MIMO Antenna Investigation

**Figure 20:**
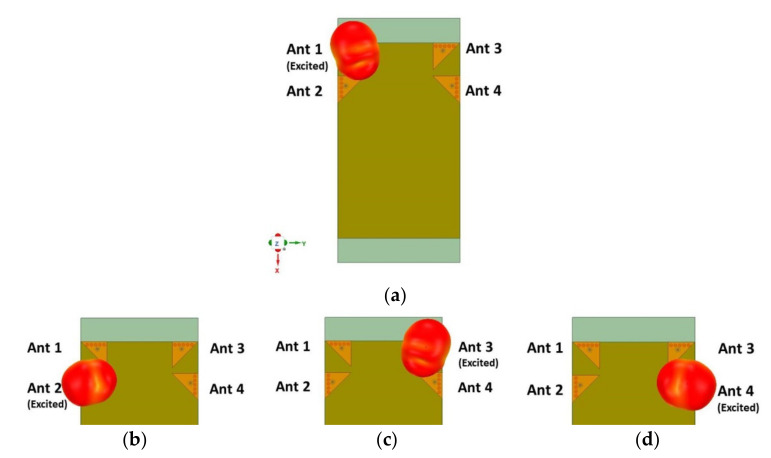
Radiation pattern (top view) of the four EMSIW MIMO antennas at 3.43 GHz when (**a**) Ant 1 is excited, (**b**) Ant 2 is excited, (**c**) Ant 3 is excited, and (**d**) Ant 4 is excited when all others are matched to 50 Ω.

Following the discussion in Section 2.3 and the validated design presented above, a four-element MIMO antenna (see Figure 13a) was studied and its MIMO diversity performance is discussed here. Figure 20 depicts the 3D radiation pattern (top view) of the four elements antenna when only one antenna is excited at a time and all others are matched. As observed in Figure 20, the pattern diversity is visible and antenna pairs, namely, Ant 1 and Ant 2 and Ant 3 and Ant 4, are orthogonally polarized.

**Figure 21:**
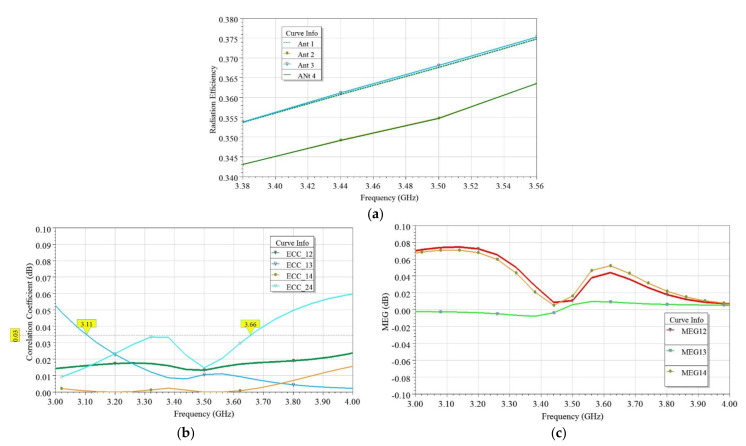
Simulated performance of four-element EMSIW MIMO antenna. (**a**) Radiation efficiencies, (**b**) envelope correlation coefficients (ECC), and (**c**) mean effective gain (MEG) variation vs. frequency.

Similarly, in Figure 21a, the radiation efficiencies of the four antennas are plotted. As observed from Figure 21a, the efficiency varied in the range of 35–38%. Table 3 summarizes the mutual diversities existing among the four elements of the MIMO antenna. Furthermore, the diversity parameter envelope correlation coefficient (ECC) was determined, as shown in Figure 21b. The calculated ECC was ≤0.03, which is much below the specified level of 0.5 [30].

More specifically, antenna pairs Ant 1 and Ant 4 and Ant 2 and Ant 3 exhibited minimum coupling due to the three diversities between them (see Table 3). Furthermore, the MEG ratio is determined [35] to quantify the power balance and diversity losses. The MEG ratios in dB of the proposed four-element MIMO antenna pairs are found below 0.1 dB (see Figure 21c), which is far below the maximum allowable of 3 dB. Therefore, low diversity losses are assured for the proposed design presented in this paper.

### 3.3. Twelve-Element MIMO Antenna Investigation

**Figure 22:**
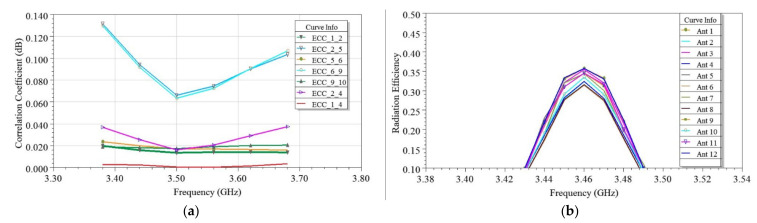
For the twelve-element EMSIW MIMO antenna, the plot of simulated (**a**) envelope correlation coefficients (ECC) and (**b**) antenna radiation efficiencies vs. the frequency.

Based on the design concept for two and four-element MIMO antennas, the twelve-element MIMO antenna was developed, as discussed in Section 2.5. An optimized antenna case A was reached after detailed analysis, as shown in Figure 1, where six EMSIW antennas were arranged on each long edge AB and CD side of the PCB. Such high-order antennas are required at the user terminal side to achieve high data rates [21,22,23,24,25,26,27,28,29]. These antennas were arranged strategically, considering various diversity techniques, as discussed in Section 2. The diversity performance, using ECC, was determined for the proposed twelve-element MIMO antenna and plotted in Figure 22a for various sets of antenna pairs, namely, Ant 1 and Ant 2, Ant 2 and Ant 4, Ant 2 and Ant 5, Ant 5 and Ant 6, Ant 6 and Ant 9, and Ant 9 and Ant 10. The computed ECC for antenna pairs were found ≤0.1, justifying good diversity performance and interference-free independent channel communication. As shown in Figure 22b, the radiation efficiency of antennas was very low, varying from 20–40% due to the use of available substrate FR4, which is lossy. However, efficiencies and gain of the proposed antenna can be improved further by using low-loss substrates such as Rogers RT Duroid [8].

### 3.4. Channel Performance Evaluation

**Figure 23:**
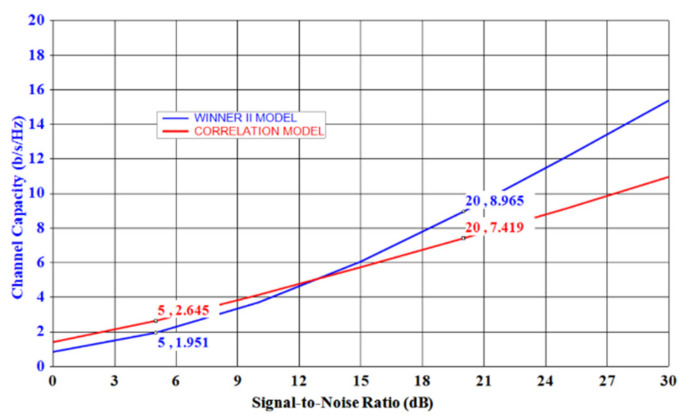
Channel capacity comparison using the two-channel models, i.e., WINNER II and CBC for the developed 2 × 2 MIMO system.

Channel performance evaluation of the developed two-element MIMO antenna and its four and twelve-element MIMO extended designs was carried out by considering the equal set of antennas at the transmitter and receiver in SystemVue Environment [37]. In this scenario, the transmitting antennas were considered uncorrelated and lossless, while the receiving antennas were the proposed antennas, which evolved as 2 × 2, 4 × 4, and 12 × 12 MIMO systems. The channel capacity in each case was investigated by considering the standard channel models, namely Winner II (WII) and correlation-based channel (CBC) models. The WII model is a geometry-based stochastic channel model that considers a channel made up of numerous clusters. Each cluster is the sum of various sub-paths. The channel impulse response was calculated in this approach, as per the details provided [38]. The CBC model combines the spatial properties of the multipath with the spatial properties of the transmitter and receiver. Subsequently, the correlation matrix is generated according to the details described [38]. The channel matrix was generated for the developed antennas in both cases and used to determine the channel capacity (CC) by averaging over 100,000 independent and identically distributed Rayleigh fading realizations when the SNR is varied from 0 to 30 dB. It was plotted for a 2 × 2 MIMO system in Figure 23. By observing the calculated CC value, as shown in Figure 22, the 2 × 2 MIMO system provides higher CC with a low SNR region (<13 dB) when the CBC model was used, however under high SNR region (>13 dB), the WII model was better than the CBC model. By taking a typical SNR = 20 dB, the ideal upper limit of CC for a 2 × 2 MIMO system was equal to 11.5 bps/Hz [21], and when the WII model was used, it delivered a peak CC of 8.965 bps/Hz when operating at 3.44 GHz. Similarly, the extended designs of 4 × 4 and 12 × 12 MIMO systems delivered a peak CC of 18.24 and 56.37 bps/Hz in the same scenario, which was 2.25 times and 6.25 times the 2 × 2 MIMO case, thus confirming a good multiplexing capability of the proposed antenna.

### 3.5. Effect of User Hands on the Antenna Performance

**Figure 24:**
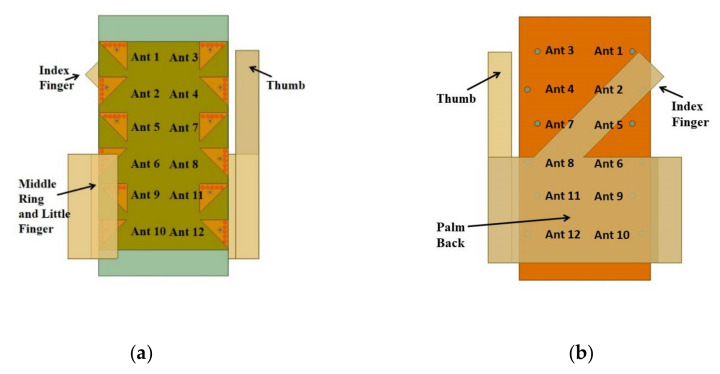

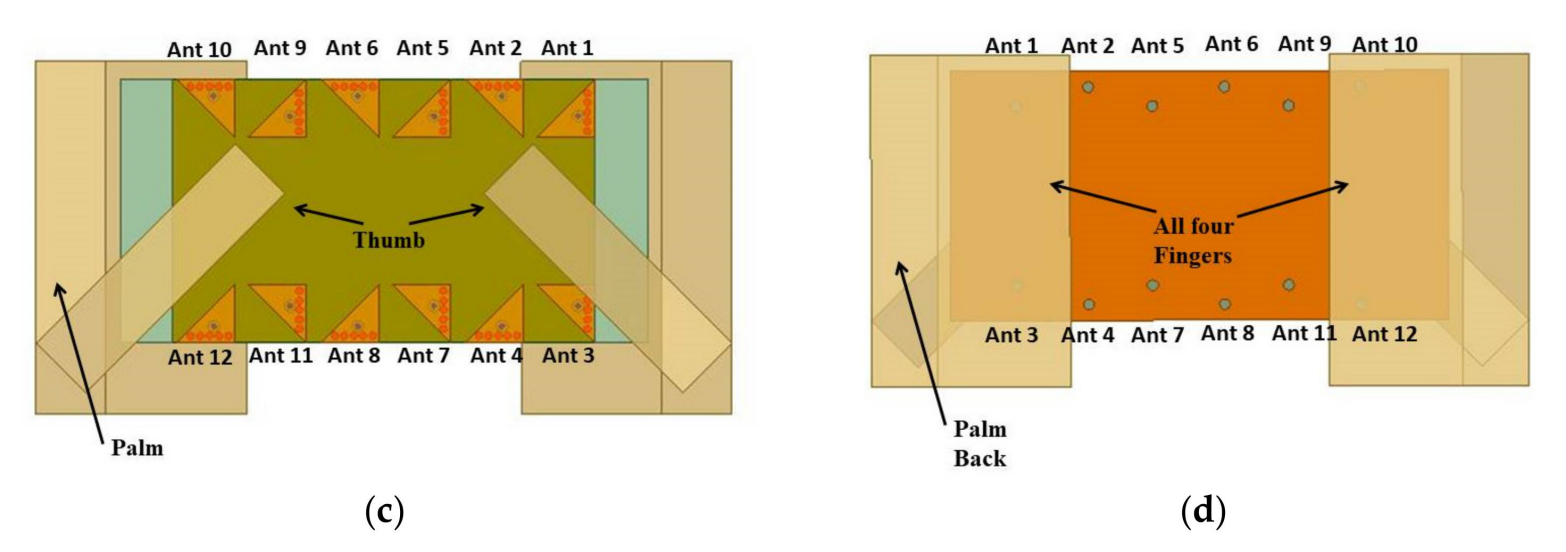
Different hand usage scenarios. (**a**) Single-hand mode (SHM) front view, (**b**) SHM back view, (**c**) dual-hand mode (DHM) front view, and (**d**) DHM back view.

In order to present the proposed study in a more realistic smartphones scenario, the antenna performances, such as S-parameters, radiation efficiencies, and ECC, were evaluated considering the effect of the user’s hand. Since the proposed design works in the Sub-6 GHz band, the analysis was restricted to data transmission mode [29], which is classified as a single-hand mode (SHM), or talk mode, and dual-hand mode (DHM), or read mode (See Figure 24). Due to computational limitations, the simplified hands’ models [39,40] were considered in the proposed study. Tissue properties [41] of the hands in the model were chosen considering the worst-case scenario (ε_r_ = 51.4 and σ = 2.56) [42] (See Table 4). In the simulation, antenna PCB was kept at a gap of 4 mm from the hand surface to account for antenna casing [39,40]. Various hands gestures scenarios are shown in Figure 24a–d.

**Figure 25:**
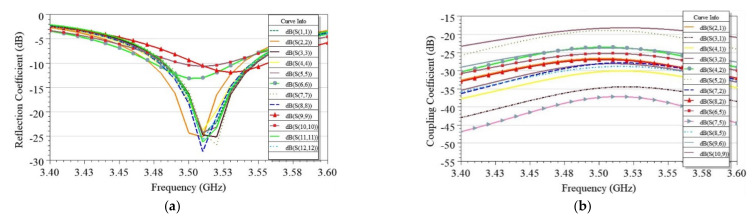

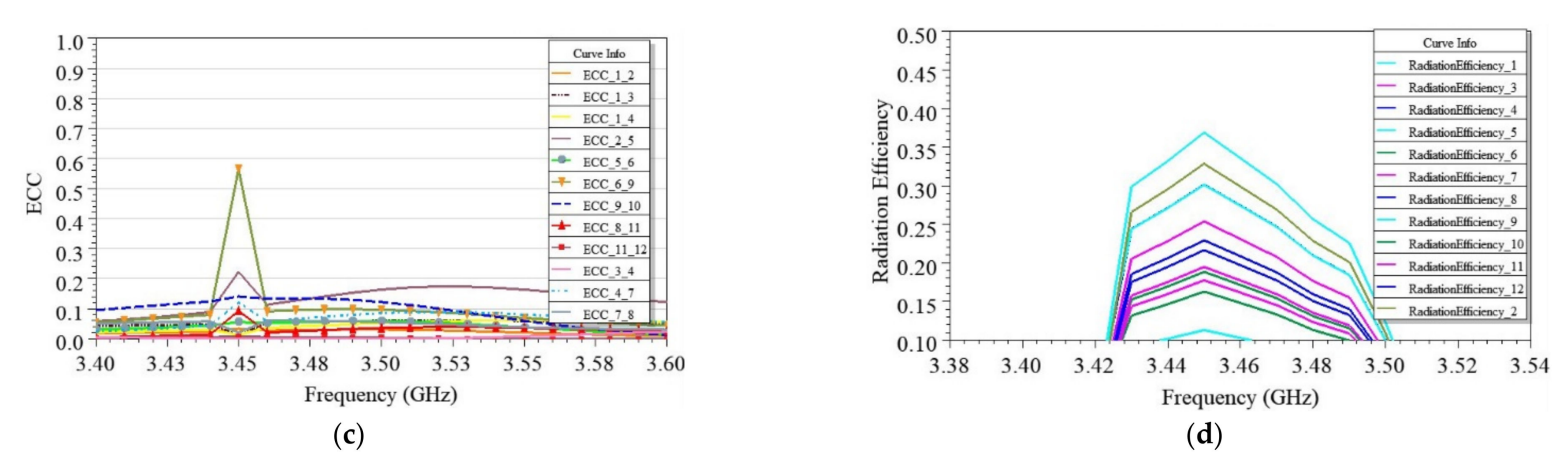
In the SHM operation simulated results for (**a**) reflection coefficients, (**b**) coupling coefficients, (**c**) envelope correlation coefficients (ECC), and (**d**) radiation efficiencies.

**Figure 26:**
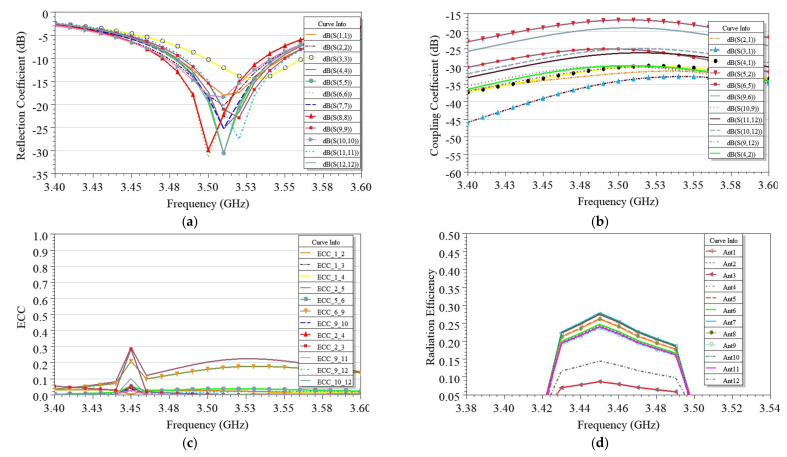
For the DHM, operation simulated (**a**) reflection coefficients, (**b**) coupling coefficients, (**c**) envelope correlation coefficients (ECC), and (**d**) radiation efficiencies.

The simulation results of the SHM and DHM operations are shown in Figure 25 and Figure 26, respectively. Various observations are noticeable from Figure 25. In the SHM case, three fingers are on the front side cover Ant 6, Ant 9, and Ant 10 (see Figure 24a); these antennas were dielectrically loaded towards the higher frequency side by the fingers. Hence, impedance matching was slightly affected. However, it could still cover the required bandwidth. All the antennas maintained isolation >18 dB and ECC < 0.2 except for antenna pairs Ant 6 and Ant 9, for which ECC rose above 0.5 limits. Most antennas’ radiation efficiencies were maintained above 20% except for Ant 9 and Ant 10, which were covered partially by hand tissues. The lossy hand tissues absorbed some portion of energy. However, the proposed MIMO system will still function with at least 20% efficiency in 5G cellular communication [23].

In the DHM case, from Figure 24c, it was observed that the right thumb was partially covering the Ant 3 on the front side. Because of this, the dielectric loading of Ant 3 is visible in Figure 26a. Nonetheless, it covered the required bandwidth at −6 dB. Furthermore, the MIMO system was working with ECC well below 0.3 and offering isolation >17 dB. In addition to that, except for Ant 3 and Ant 12, all antennas delivered at least 20% efficiency. Hence, it was clear from the discussion that in both SHM and DHM operation, the proposed antenna design had no significant user hand effects, thus making it suitable for smartphone use.

### 3.6. Specific Absorption Rate (SAR) Analysis

**Figure 27:**
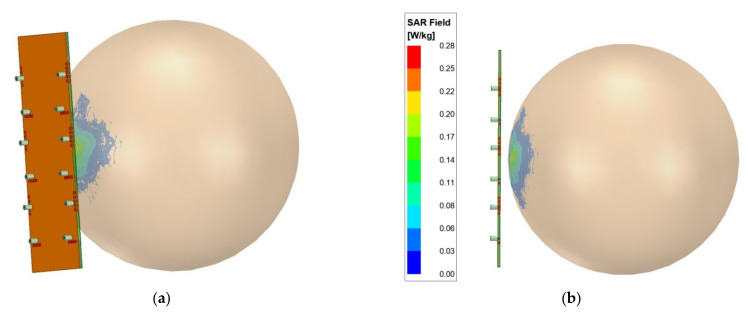
SAR investigation of the proposed twelve-element EMSIW MIMO antenna: (**a**) antenna in the vicinity of the phantom head model (trimetric view), (**b**) SAR field variation inside homogenous head phantom model at 3.5 GHz (side view).

SAR is used to characterize electromagnetic radiation exposure to humans from non-ionizing radiations. Standard threshold limits are defined by the ICNIRP [43] organization, which should not cross 1.6 W/Kg for 1-g tissue and 2 W/Kg for 10-g tissue [28]. It signifies the radiation energy absorbed by the human tissues. Therefore, wireless antenna systems must comply with these guidelines. In the proposed work using Ansys HFSS, the twelve-element MIMO antenna is simulated to determine the specific absorption rate. In order to calculate the SAR, the antenna is placed closed to human body parts models, such as the head and hand, which could be homogenous or heterogeneous [40,44,45]. The one-layer homogeneous models may not be good, and the design of multilayer, e.g., 6-layers models, is a better choice [44]; however, considering computational limitation existing at the authors’ institute, the present study was restricted to one homogeneous layer model. Hence, in this work, a worst-case scenario [42] for tissue parameters [41] was selected from Table 5, and a spherical-shaped head phantom (εr = 64.53 and σ = 4.6 @ 3.5 GHz) was used [40,45]. A cubical model was not considered, as it may cause antenna loading [45]. The antennas in the proposed study were kept at a distance of 5 mm (to account for ear pinna) from the head phantom with a radius of 80 mm, and all the 12 ports of the antennas were excited with an input power of 25 mW, each with the same phase. The placement of the antenna near the head phantom is shown in Figure 27a. The calculated SAR is plotted in Figure 27b, which shows a peak of 0.28 W/kg. Hence, the proposed antenna in this work operated within the safe limit of the mobile phone user.

### 3.7. Performance Comparison

Table 6 shows the comprehensive comparison between the proposed antenna array designs and the recently reported works on SIW MIMO antennas [5,17,18] as well as the 5G handset MIMO antennas [21,22,23,24,25,26,27,28,29]. The [5,17] and [18] are the EMSIW MIMO antennas, as in the proposed work. However, these works [5,17,18] are not focused explicitly on the smartphone scenario. Comparing the two-element MIMO antenna developed in this work and reported in [5,18] confirms that the proposed design is more compact in terms of the area required by the unit element, along with improved bandwidth and ECC. In addition to that, the proposed design achieved high isolation in the adjacent antennas scenario compared to [5] without using any decoupling network (DN) with the help of proper antenna placement to achieve multiple diversity. Although [17,18] also did not use any DN, the proposed design achieved higher isolation as compared to [17,18]. The reason is the same, i.e., implementation of multiple diversities in the proposed design. However, the lower antenna efficiency and gain in the proposed design could be improved by using a low-loss dielectric substrate [8]. Furthermore, in the future, creating the cavity in the substrate in the proposed antenna could improve the performance [46]. The increased levels of cross-polar components can be reduced by suppressing higher-order modes [47]. Moreover, [17] is a more compact design; however, its gain is very low, making it a poor candidate for 5G NR. Therefore, comparing the presented two-element design with previous similar EMSIW work establishes that the proposed design overall is much better.

To further demonstrate that the proposed twelve-element MIMO antenna design is improved, a comparison was carried out with recently published 5G smartphone MIMO antennas [21,23,24,25,26,27,28,29]. The proposed design is compact and straightforward in terms of unit antenna area as compared to [21,23,24,25], as the author in [23] used DN to improve the isolation, but isolation achieved was lower than reported in this paper. Additionally, the proposed work was performed using SIW technology. Therefore, it gives the design an edge over others in terms of power handling and easy integration with radiofrequency circuits. Although work reported in [26,27,28,29] showed more compact MIMO antennas exhibiting good radiation characteristics with improved bandwidths, the proposed high-order MIMO antenna reported in this paper showed comparable gain and ECC with even better isolation; however, these performances could be further improved [14,16]. In addition to that, channel capacity observed in the proposed 12 × 12 MIMO system was found to be nearly 6.25 times higher than the upper limit for a 2 × 2 MIMO system. Moreover, strategically placing the antennas on PCB such that avoiding corners of PCB made the proposed design negligibly affected by the user’s hands in both the SHM and DHM operations. Hence, the detailed investigations of the proposed high-order MIMO antenna justify its suitability for 5G NR.

## 4. Conclusions

The proposed twelve-element MIMO antenna was thoroughly investigated for 5G NR. The unit element in the MIMO antenna was the EMSIW cavity resonator. A two-element MIMO antenna based on a compact EMSIW cavity resonator was designed and developed to operate at 3.5 GHz, and its performances were evaluated through both comprehensive parametric simulations and measurement. Multiple diversity techniques were utilized to achieve good multiplexing performance. It offered a −6 dB bandwidth of 250 MHz with a peak gain of 3.4 dB and significant isolation of ≥35 dB. The measured co- and cross-field patterns in both the planes were found in closed conformity with simulated results. Moreover, after design validation, the design concept was extended to develop four-element and twelve-element MIMO antennas. Proper placement of closely spaced antennas generated orthogonal polarization, pattern, and spatial diversity that helped achieve high isolation ≥22 dB without using any complex decoupling network, making the proposed design simple and low cost. Furthermore, experimentally confirmed results suggest low ECC (<0.13) and MEG within ±1 dB; thus, confirming the antenna suitability to perform under fading channel environment with good multiplexing capability. The channel capacity performance of the proposed antenna was extensively evaluated using Winner II and CBC channels models under the SystemVue environment. The peak channel capacity in the 12 × 12 MIMO system reached 6.25 times the upper limit for 2 × 2 MIMO. Moreover, no significant deterioration was found in the presence of the user’s hands, and SAR was obtained within safe limits. Thus, the extensive results presented and detailed comparisons in this paper justify the suitability of the proposed MIMO antenna for 5G new radio.

## Figures and Tables

**Table 1:** Optimized dimensions of the FMSIW cavity.

Parameter	Value (mm)	Parameter	Value (mm)
*L*	46.4	*a*	30
*W*	35	*s*	3
*W_f_*	3	*d*	2
*L_f_*	11.4	*h*	1.6
*L_in_*	6	*t*	0.035
*W_in_*	0.3		

**Table 2:** Different types of diversity existing in two-element MIMO antenna for the adjacent scenario.

Type of Diversity	Case 2.a	Case 2.b	Case 2.c	Case 2.d
Pattern	No	No	Yes	Yes
Polarization	No	No	Yes	Yes
Spatial	No	No	No	No

**Table 3:** Mutual diversities among antennas of four-element MIMO system.

Antenna	1	2	3	4
**1**	NA	P/Po	P/S	P/Po/S
**2**	P/Po	NA	P/Po/S	P/S
**3**	P/S	P/Po/S	NA	P/Po
**4**	P/Po/S	P/S	P/Po	NA

Abbreviations: NA = Not applicable, P = pattern diversity, Po = polarization diversity, S = spatial diversity.

**Table 4:** Different tissue properties [41] of hand model, considered at 3.5 GHz simulation study.

Tissue	Thickness (mm)	Permittivity	Conductivity (S/m)
Skin	2	37	2.02
Muscle	2	51.4	2.56
Bone	15	10.8	0.615
**Worst Case**	**20**	**51.4**	**2.56**

**Table 5:** Different tissue properties [41] of the head phantom model at 3.5 GHz.

Layer Order	1	2	3	4	5	6	7	
**Tissue**	White Matter	Gray Matter	Cerebrospinal Fluid (CSF)	Skull Inner	Skull Cancellous	Skull Outer	Skin	**Worst Case**
**Relative** **Permittivity**	34.97	40.68	64.53	10.77	17.4	10.77	36.98	**64.53**
**Bulk Conductivity (siemens/m)**	1.825	2.38	4.6	0.62	1.2	0.62	2.04	**4.6**

**Table 6:** Performance comparison of the proposed MIMO antenna designs with previous works.

Reference	Technology Used	MIMO Order	Substrate Used	Unit Antenna Area (λ_o_^2^)	Bandwidth (MHz) −10 dB/−6 dB	Gain (dBi)	Minimum Efficiency (%)	Isolation (dB)	DT Used	ECC	PCC @ 20 dB SNR (bps/Hz)
This work	EMSIW	2 (Fab.) 4 (Sim.) 12 (Sim.)	FR4 (1.6 mm)	0.0171	100/250 80/150 80/120	3.4 (peak)	35 (sim.) 35 (sim.) 20 (sim.)	≥35 ≥25 ≥22	No No No	≤0.013 ≤0.04 ≤0.13	8.96 18.24 56.37
[5]	EMSIW	2 (Fab.) 4 (Sim.)	RT/duroid 5880 (1.57 mm)	0.0437	120/− 80/−	4.2 (peak) 4.03 (peak)	NR NR	≥30 ≥18	Yes Yes	≤0.1 NR	NR NR
[17]	EMSIW	4 (Fab.)	FR4 (1.6 mm)	0.0144	47/−	−3 (peak)	NR	≥20	No	≤0.03	NR
[18]	EMSIW	2 (Fab.)	F4B2 (3 mm)	0.0231	60/−	4.6 (peak)	70 (Meas.)	≥18.5	No	≤0.04	NR
[21]	Microstrip	8 (Fab.)	FR4 (0.8 mm)	0.0408	200/−	NR	62 (Meas.)	≥17.5	No	≤0.05	40.8
[23]	Microstrip	2 (Fab.) 8 (Fab.)	FR4 (1.6 mm)	0.1225	400/600	3	80 (Meas.)	≥18	Yes	≤0.012	NR
[24]	Microstrip	8 (Fab.)	FR4 (5 mm)	0.0183	310/−	2	40 (Sim.)	≥16	No	≤0.18	NR
[25]	Microstrip	4 (Fab.)	FR4 (1.6 mm)	0.0466	−/350 ^a^	6 (peak)	75 (Meas.)	≥17	No	≤0.05	NR
[26]	Microstrip	8 (Fab.)	FR4 (1.6 mm)	0.0191	−/350 ^a^	4.5 (peak)	50 (Meas.)	≥11	No	≤0.01	NR
[27]	Microstrip	10 (Fab.)	FR4(0.6 mm)	0.0035	500/−	4 (peak)	60 (Meas.)	≥12	No	≤0.02	42
[28]	Microstrip	10 (Fab.)	FR4(0.8 mm)	0.0144	−/400	4 (peak)	50 (Sim.)	≥15	No	≤0.1	38.1
[29]	Microstrip	10 (Fab.)	FR4(0.8 mm)	0.0066	−/400 ^a^	>5.3	83 (Meas.)	≥20	No	≤0.06	41

Abbreviations: Fab. = fabricated, Sim. = simulated, Meas. = measured, DT = decoupling technique, PCC = peak channel capacity, NR = not reported. ^a^ For 5G Lower Band.

## Data Availability

Not applicable.
